# Unveiling the hidden link: diabetes mellitus as a catalyst for orbital apex syndrome

**DOI:** 10.3389/fendo.2026.1770045

**Published:** 2026-02-25

**Authors:** Shu Xin Gao, Jie Gao

**Affiliations:** General Practice Department, Cangzhou Central Hospital, Cangzhou, China

**Keywords:** diabetes mellitus, diabetic fungal infections, diabetic microvascular damage, orbital apex syndrome, pathophysiological mechanisms

## Abstract

**Background:**

Orbital Apex Syndrome (OAS) is a disease of multiple brain nerves at the orbital apex leading to vision loss and neurological impairments. Diabetes Mellitus (DM), a metabolic disorder with cardiovascular, immunological and neurological effects, is involved in OAS pathogenesis. However, the association between DM and OAS is not well studied. DM and OAS are poorly understood and may not be diagnosed correctly, especially when outbreaks such as COVID-19 are being investigated.

**Methods:**

A systematic review of 33 studies published between 2000 and 2025 was conducted to analyze DM-related OAS epidemiology, pathophysiology, clinical phenotypes, and treatment outcomes, focusing on the mechanistic links, pandemic trends, and glycemic control effect on therapeutic effectiveness.

**Results:**

Chronic hyperglycemia induced orbital apex microvascular damage (endothelial dysfunction, thrombosis, vascular senescence), immunosuppression induced opportunistic infections (mostly mucormycosis), and diabetic neuropathy induced neuromuscular dysfunction. During COVID-19, diabetic patients had the highest OAS incidence (more than 70% of cases involved rhino-orbital mucormycosis). Optimal glycemic control is associated with a 32% higher antifungal treatment effectiveness and a 28% lower rate of surgical complications. Epidemiological data showed that DM was the main predisposing factor, with 71.4%–81.8% infectious OAS cases occurred in diabetic populations.

**Conclusion:**

DM is underreported as a critical catalyst for OAS with complications directly increasing severity and progression. Routine DM screening (e.g., glycated hemoglobin monitoring) and integrated glycemic management are essential for OAS prevention and treatment. Long-term studies on inflammatory factors and personalized multidisciplinary care are needed to address mechanistic gaps and improve visual and neurological outcomes in high-risk diabetic patients.

## 1 Introduction

OAS refers to a constellation of symptoms and signs which result from the involvement of various structures in the region of the orbital apex by a disease process. These structures include the four rectus muscles taking their origin from the tendinous annulus of Zinn, the optic nerve and ophthalmic artery through the optic canal. The superior and inferior branches of the oculomotor nerve, the abducens nerve and the naso-ciliary nerve pass through the annulus of Zinn through the middle portion of the superior orbital fissure (1). It is a rare but severe disease of the orbital apex, caused by orbital apex lesions that affect multiple cranial nerves (including the optic nerve and oculomotor nerve), characterized by visual loss, ophthalmoplegia and periorbital pain (2). Although OAS is varied (infections, tumors, trauma), recent studies have found that DM, a chronic metabolic disorder that affects 463 million adults globally (3). DM-related complications (microvascular damage, immune dysregulation) increase OAS rate and worsen the disease (4, 5).

Although more clinical studies link DM to OAS are emerging, most studies focus on single center case reports or specific diseases (mucormycosis, for example), without systematic integration of epidemiology, mechanistic pathways, and clinical management. This limits clinicians’ ability to detect, diagnose and treat DM-related OAS timely. In this review we summarize 2015–2025 literature to clarify the relationship between DM and OAS, translating results into actionable clinical guidance, aligning Science Progress’s mission to bridge basic research and clinical practice.

## 2 Literature search and selection

Literature on DM-related OAS was searched in PubMed (2000–2025) using relevant keywords. Studies addressing core evidence and contextual background were included, with non-DM/OAS literature excluded. Thirty-three eligible studies (retrospective cohorts, case-control, systematic reviews, case reports, thematic reviews) were selected, prioritizing 2014–2025 publications (31/33); 2 seminal older studies (6, 7) were retained for important value.

## 3 Diabetes-related OAS: epidemiological characteristics

The pathophysiological links between DM and OAS (e.g., vascular damage, immunosuppression) are reflected in consistent epidemiological patterns, with DM emerging as a dominant risk factor across populations and clinical scenarios. This section integrates data on overall incidence, pandemic-era trends, and regional variations to contextualize the burden of DM-related OAS.

### 3.1 Overall incidence in diabetic populations

Numerous studies indicate that diabetic patients are more likely to develop OAS than non-diabetic patients. In a retrospective study of 28 mucormycosis patients (a major infectious cause of OAS), 20 patients (71.4%) had DM (85% of patients had OAS symptoms (e.g., vision loss, ophthalmoplegia) (8). In another study of 11 rhino-orbito-cerebral mucormycosis patients first presented with OAS, 9 patients (81.8%) had type 2 DM (T2DM), consistent with DM being a key predisposing factor (4). Even non-infectious OAS (e.g., inflammation) was associated with a 3.2-fold higher risk of DM onset attributable to chronic inflammation and vascular dysfunction (9). Although this trend is grounded in observational data rather than causal evidence.

### 3.2 Pandemic-era surge: COVID-19 as a co-trigger

The COVID-19 pandemic increased the DM-OAS association due to pandemic-related immunosuppression (e.g., corticosteroid use) and worsening DM control. In a study of 11 severe COVID-19 patients with ROM, 8 (72.7%) had uncontrolled type 2 diabetes mellitus(T2DM) and 7 (63.6%) developed to OAS (10). In a larger Indian cohort of 60 COVID-19 related ROCM patients, 59 (98.3%) had DM, and 42 (72.2%) had OAS-related ocular manifestations (e.g. optic neuropathy, ptosis) (11). These results suggest that infectious outbreaks could synergize with DM to increase OAS risk. It is important to monitor diabetic COVID-19 survivors.

### 3.3 Regional and population-specific trends

Regional variation in DM prevalence and access to healthcare further shape OAS prevalence. In subtropical Australia, 30 adult orbital fungal disease patients reported 33.3% of OAS (DM is second most common risk factor after hematological diseases) (12). In India (T2DM prevalence >11%), an analysis of 11 ROCM patients demonstrated a 100% incidence of OAS (81.8% in the primary analysis cohort) (13). These regional differences highlight context-specific prevention strategies (e.g., enhanced DM control in high-prevalence regions).

## 4 Pathophysiological mechanisms linking DM to OAS

DM drives OAS pathogenesis via three interconnected, mutually reinforcing pathways: microvascular damage, immune dysregulation, and neuropathy. These pathways converge to disrupt orbital apex homeostasis, leading to nerve dysfunction and clinical OAS.

### 4.1 Microvascular damage: the vascular basis of OAS

Chronic hyperglycemia is the root cause of DM-induced microvascular dysfunction, which directly impairs orbital apex blood supply. Hyperglycemia damages vascular endothelial cells, promotes thrombus formation, disrupts blood supply to the orbital apex, and causes nerve ischemia and hypoxia, leading to neurological dysfunction and inducing OAS (14). Key mechanisms include:

Endothelial dysfunction: Hyperglycemia activates the polyol pathway and advanced glycation end product (AGE) formation, leading to oxidative stress and diminished NO bioavailability (15). This leads to endothelial relaxation, vascular constriction and decreased flow to orbital apex, the region with a fragile nerve network.

Inflammatory vascular senescence: The NLRP3 inflammasome (a key mediator of inflammation) links DM to vascular aging: activated NLRP3 triggers IL-1β and IL-18 release, accelerating endothelial senescence and vascular stiffness (16). In orbital apex tissues, this senescence disrupts blood-nerve barrier integrity, facilitating edema and nerve compression.

Thrombosis risk: DM-induced platelet hyperactivity and reduced fibrinolysis increase thrombus formation in orbital apex vessels (3). Thrombosis further reduces blood supply, leading to nerve ischemia-hypoxia and irreversible damage (e.g., optic nerve atrophy).

### 4.2 Immune dysregulation: enhancing infectious susceptibility

DM compromises both innate and adaptive immunity, increasing susceptibility to opportunistic infections— the most common cause of DM-related OAS. Key defects include:

Neutrophil dysfunction: High blood sugar reduces white blood cells’(neutrophils) ability to find, attack, and kill fungi like Mucor spp. and Aspergillus spp (5). In a case study, a 59-year-old uncontrolled T2DM patient developed OAS due to a fungal nasal septum abscess (caused by Scedosporium apiospermum)—likely due to failed neutrophil-mediated pathogen clearance (17).

Immune cell imbalance: DM shifts the immune response toward a pro-inflammatory (Th17-dominant) state, while reducing regulatory T cell (Treg) function (18). It causes an overactive inflammatory response while weakening the body’s natural “damage-control” cells. This imbalance exacerbates orbital apex inflammation, accelerating tissue damage and nerve impairment.

Mucosal barrier disruption: Hyperglycemia weakens sinus and orbital mucosal barriers, allowing pathogens to invade the orbital apex. For example, ROM (a common OAS trigger) spreads from sinus mucosa to the orbital apex via this disrupted barrier—with DM doubling the risk of invasive spread (10).

### 4.3 Diabetic neuropathy: direct nerve impairment

DM-induced neuropathy independently contributes to OAS by damaging cranial nerves in the orbital apex (19). Mechanisms include:

Axonal degeneration: Chronic hyperglycemia disrupts nerve axonal transport and energy metabolism, leading to axonal loss in the optic nerve and oculomotor nerves (3). This manifests as visual decline and ophthalmoplegia—core OAS symptoms.

Sympathetic fiber damage: DM impairs sympathetic innervation of the orbital apex, altering pupillary light reflexes. A case report of diabetic OAS patients found 78% had abnormal pupillary responses (e.g., reduced light reflex), linked to sympathetic fiber compression and neuropathy (20).

## 5 Clinical phenotypes and diagnostic strategies for DM-related OAS

DM modifies OAS clinical presentation (e.g., severity, progression speed) and requires tailored diagnostic approaches to ensure timely intervention. This section outlines key clinical features and a stepwise diagnostic framework to guide clinical practice.

### 5.1 Clinical phenotypes: DM-specific manifestations

Diabetic OAS patients exhibit distinct symptoms, driven by the underlying mechanisms of infection and neuropathy:

Ocular symptoms: Visual impairment is most common (64–85%), ranging from mild blurred vision to complete blindness (2, 13). Ophthalmoplegia (restricted eye movement, diplopia) affects 73–81%, often due to ocular motor nerve damage (4, 20). Additional ocular signs include proptosis (32%), ptosis (45%), and abnormal pupillary reflexes (78%) (2, 20).

Systemic symptoms: Fever (38–40 °C) is common in infectious OAS (e.g., mucormycosis), correlating with poorer prognosis—43% of febrile diabetic OAS patients died, compared to 12% of afebrile patients (5). Fatigue and weight loss may also occur due to uncontrolled infection and hyperglycemia.

Rapid progression: DM accelerates OAS onset to symptom peak (median 5 days *vs*. 12 days in non-diabetic patients), likely due to unchecked infection and nerve damage (10, 11).

### 5.2 Stepwise diagnostic framework

A comprehensive diagnostic approach for DM-related OAS integrates clinical history, imaging, and laboratory tests—with a focus on identifying DM status and infection etiology:

Step 1: Clinical History and Initial Assessment

DM screening: Query DM history (type, duration, medication use) and measure fasting blood glucose (FBG) and glycated hemoglobin (HbA1c). HbA1c >7% indicates poor glycemic control—a key risk factor for severe OAS (3).

Symptom timeline: Document onset speed (rapid progression suggests infection) and symptom sequence (e.g., sinus pain followed by visual loss points to ROCM).

Step 2: Imaging Examinations

CT scan: First-line imaging for bone and sinus evaluation. DM-related infectious OAS often shows sinus opacification with abnormal density extending to the orbital apex (21). For example, a 53-year-old T2DM patient with Aspergillus-induced OAS had CT findings of sinusitis and orbital apex soft tissue thickening (2).

MRI: Gold standard for soft tissue and nerve assessment. T1-weighted contrast MRI reveals orbital apex lesions (e.g., inflammation, abscesses) and nerve compression. The “track sign” (linear enhancement from sinus to orbital apex) is pathognomonic for mucormycosis-related OAS (13). Functional MRI (fMRI) may further assess nerve viability, guiding treatment decisions. For instance, in a case report, MRI identified an asymmetric contrast-enhanced lesion in the patient’s orbital apex, aiding in diagnosing OAS (22).

Step 3: Laboratory and Microbiological Tests

DM monitoring: FBG (>7 mmol/L) and HbA1c (>7%) confirm poor glycemic control.

Infection diagnosis: For suspected infectious OAS, collect sinus secretions or tissue biopsies for smear, culture, and PCR. Mucor and Aspergillus are the most common pathogens—identified in 69% and 21% of diabetic OAS cultures, respectively (21).

Inflammatory markers: C-reactive protein (CRP >10 mg/L) and procalcitonin (>0.5 ng/mL) indicate active inflammation, aiding in severity assessment (5).

Step 4: Differential Diagnosis

Distinguish DM-related OAS from similar conditions:

Cavernous sinus syndrome: Affects cranial nerves III, IV, VI, and V1/V2 but spares the optic nerve (no visual loss).

Superior orbital fissure syndrome: No optic nerve involvement (normal visual acuity) and no sinus lesions on CT/MRI.

### 5.3 Clinical practice recommendations

For clinical practice, diabetic patients with poor glycemic control (HbA1c >7%) and periorbital pain or visual changes should undergo urgent eye and imaging tests to rule out OAS. A multidisciplinary team (MDT) consultation (endocrinology + ophthalmology + otolaryngology) should be initiated within 48 hours of suspected OAS for optimal treatment coordination.

## 6 Treatment and management strategies for DM-related OAS

Effective management of DM-related OAS requires a dual focus: controlling DM and treating OAS (e.g., infection, inflammation). MDT approach is critical to balance glycemic control, anti-infective therapy, and surgical intervention.

### 6.1 Glycemic control: the foundation of treatment

Optimal glucose management improves immune function and reduces infection severity:

Acute phase: Use insulin therapy to achieve FBG 4.4–7.0 mmol/L and HbA1c <7% (3). In a retrospective case series of 28 diabetic OAS patients, insulin-treated patients were reported to have a 32% higher antifungal response rate and a 28% lower incidence of surgical complications. These numerical trends represent an observational association, and confounding factors (e.g., timing of surgical intervention, baseline immune status) may contribute to the outcomes; causal effects of insulin therapy on treatment efficacy remain unvalidated (8).

Chronic phase: Transition to oral hypoglycemics (e.g., metformin, SGLT2 inhibitors) or long-acting insulin, avoiding glucocorticoids unless absolutely necessary for inflammation control (due to hyperglycemia risk).

### 6.2 Targeted treatment of OAS etiologies

Infectious OAS (Most Common):

Antifungal therapy: Amphotericin B (0.7–1.0 mg/kg/day IV) is the first-line treatment for mucormycosis; posaconazole (400 mg twice daily) or isavuconazole (200 mg once daily) are recommended as salvage therapies (21, 23, 24). Isavuconazole has key advantages over other triazoles: fewer drug interactions, lower organ toxicity, and dual intravenous/oral formulations for flexible use (24). Treatment duration is 6–12 weeks, guided by clinical response and imaging follow-up. Novel antifungal agents may expand therapeutic options for difficult-to-treat cases. Novel antifungal drugs that are currently in phase II/III clinical trials include some first-in-class molecules (fosmanogepix, olorofim, ibrexafungerp) and modified compounds from existing antifungal drug classes with improved pharmacologic properties (rezafungin, tetrazoles, encochleated amphotericin B) (25). Among the first-in-class antifungals, only fosmanogepix displays some effect against Mucorales. Fosmanogepix is an inhibitor of the glycosylphosphatidylinositol (GPI) biosynthesis pathway, which has a potent fungistatic effect with broad spectrum activity against most pathogenic yeasts and molds (26).

Surgical intervention: Endoscopic sinus surgery (ESS) removes infected tissue and relieves orbital apex pressure—85.7% of diabetic OAS patients had symptom improvement after ESS (27). For severe mucormycosis, orbital exenteration may be necessary to prevent intracranial spread, though this is a last resort (5).

Non-Infectious OAS (Inflammation, Trauma):

Glucocorticoids: Low-dose methylprednisolone (0.5 mg/kg/day) reduces inflammation, but only with strict glucose monitoring (e.g., hourly FBG checks) (28).

Immunosuppressants: Azathioprine (1–2 mg/kg/day) may be used for autoimmune-related OAS, though data in diabetic patients is limited (29).

### 6.3 MDT collaboration: key to success

Managing orbital apex syndrome (OAS) in patients with diabetes requires a multidisciplinary approach, presenting several challenges. OAS necessitates the collaboration of ophthalmology, otolaryngology, and neurology. However, effective communication among these disciplines is often hindered in clinical practice, causing delays in diagnosis and treatment. For instance, ophthalmologists might prioritize ocular symptoms, whereas otolaryngologists may focus on sinus lesions. This lack of communication can lead to an incomplete assessment of the patient’s condition (30). An MDT team ensures coordinated care:

Endocrinologist: Manages glycemic control and adjusts DM medications.

Ophthalmologist: Monitors visual function and performs orbital procedures (e.g., decompression).

Otolaryngologist: Performs ESS and sinus debridement.

Infectious disease specialist: Guides antifungal therapy and monitors for drug toxicity.

Example MDT protocol: A 62-year-old T2DM patient with mucormycosis-related OAS underwent ESS (otolaryngology), insulin therapy (endocrinology), and amphotericin B (infectious diseases)—resulting in visual recovery from 20/200 to 20/60 (31).

## 7 Controversies, challenges, and unresolved issues

Despite progress in understanding DM-related OAS, several gaps remain, limiting optimal clinical care.

### 7.1 Controversies in treatment efficacy

Antifungal resistance: Emerging reports of Mucor resistance to amphotericin B in diabetic patients raise concerns—32% of resistant cases failed treatment, compared to 8% of susceptible cases (5). The role of combination antifungal therapy (e.g., amphotericin B + posaconazole) remains unproven and requires randomized controlled trials.

Glucocorticoid use: While glucocorticoids reduce inflammation, they worsen hyperglycemia and may enhance fungal growth. No consensus exists on the “safe” dose or duration in diabetic OAS patients.

### 7.2 MDT coordination challenges

In clinical practice, poor communication between specialties delays diagnosis: 45% of diabetic OAS patients waited >7 days for MDT consultation, leading to irreversible visual loss (9). Barriers include fragmented electronic health records and lack of standardized MDT referral protocols.

### 7.3 Unresolved mechanistic gaps

Immune regulation: The precise role of inflammatory factors (e.g., IL-6, TNF-α) in DM-OAS remains unclear. No studies have measured orbital apex tissue cytokine levels in diabetic patients to confirm their pathogenic role. Rare pathogens: Diagnostic and treatment strategies for OAS caused by rare fungi (e.g., Scedosporium apiospermum) are limited, with only 5 case reports published since 2015 (17). A key limitation of this review is the constraints of the existing evidence base for diabetes mellitus (DM)-related orbital apex syndrome (OAS). First, most data derive from small cohorts and case series (e.g., 11 patients (4), 28 patients (8), 60 patients (11)), with sample sizes limiting statistical power and generalizability to broader populations. Second, evidence is geographically concentrated—disproportionately from regions with high DM and mucormycosis prevalence (China (4), India (11, 13), subtropical Australia (12))—with sparse data from Europe, North America, and Africa, potentially obscuring population-specific trends. Third, the literature is heavily weighted toward infectious etiologies, particularly mucormycosis (5, 21), with limited evidence on non-infectious DM-related OAS (e.g., inflammatory, vascular etiologies), making conclusions on non-infectious forms preliminary. Finally, all findings rely on observational designs (retrospective cohorts, case series), which identify associations but not causal relationships—confounding factors (e.g., timely surgical debridement, pathogen virulence) may influence trends. Future prospective studies with larger, geographically diverse samples and inclusion of non-infectious etiologies are needed to validate current findings.

## 8 Future research directions

Future advancements are anticipated in the diagnosis and treatment of orbital apex syndrome and diabetes. Diagnostic methods are expected to become more precise and rapid. For instance, molecular biology techniques may enhance pathogen detection, facilitating early diagnosis and targeted treatment (1). To address current gaps, future research should focus on three priority areas:

### 8.1 Mechanistic studies

longitudinal cytokine profiling: Measure serum and orbital apex tissue levels of IL-6, TNF-α, and NLRP3 in diabetic OAS patients to identify predictive biomarkers of severity.

Gene editing for immune restoration (experimental future research): Gene editing technologies (including CRISPR-Cas9) remain in the preclinical, experimental stage, with no clinical application in diabetic OAS patients to date. Preclinical studies in diabetic animal models have explored repairing immune-deficient genes (e.g., NLRP3) to enhance antifungal immunity (32), but this research is purely exploratory and has not been translated into clinical strategies for OAS. This area represents a long-term future research direction rather than an emerging clinical approach (6).

### 8.2 Diagnostic innovations

Molecular pathogen detection: Point-of-care PCR tests for rare fungi (e.g., Scedosporium) could reduce diagnostic time from 72 hours to <4 hours (1).

fMRI for nerve viability: Functional MRI (e.g., diffusion tensor imaging) may predict visual recovery, guiding surgical decisions—currently, no validated imaging biomarkers exist.

### 8.3 Personalized treatment

Developing precise personalized treatment plans is an urgent issue in medical treatment. Patients vary in their conditions, physical states, and responses to therapies. Future research should focus on formulating optimal treatment plans that consider these factors to enhance treatment efficacy and improve patients’ quality of life (33). Stratified therapy: Develop algorithms based on DM type (T1DM *vs*. T2DM), HbA1c, and pathogen to tailor antifungal and surgical approaches. For example, T1DM patients with HbA1c >9% may require more aggressive insulin therapy and earlier surgery.

Long-term prognosis studies: Conduct 5-year follow-up of diabetic OAS patients to assess visual recovery, OAS recurrence, and the impact of DM complications (e.g., retinopathy) on outcomes (7).

## 9 Conclusion

Diabetes Mellitus is a critical, underrecognized catalyst for Orbital Apex Syndrome, driving pathogenesis via microvascular damage, immune dysregulation, and neuropathy. Epidemiological data confirm DM elevates OAS risk—particularly during infectious outbreaks like COVID-19—while clinical evidence highlights the need for integrated DM management and targeted OAS treatment.

The overview summarizes what clinicians need to do: Screen all OAS patients for DM, conduct stepwise diagnostic evaluations (including history taking and imaging tests), and MDT care to balance glycemic control with OAS treatment. Future work should focus on mechanistic biomarkers, diagnostic innovations, and personalized care for OAS patients.

By bridging the gap between DM and OAS research, this work aligns with Science Progress’s goal of translating cross-disciplinary findings into clinical practice—ultimately reducing the burden of this severe, preventable condition.
